# Baseline Plasma GPX3 Level Predicts Efficacy of Insulin-Sensitization Drug Chiglitazar in Type 2 Diabetes

**DOI:** 10.1007/s43657-025-00266-1

**Published:** 2025-07-29

**Authors:** Zehui Cao, Haonan Chen, Qiaochu Chen, Chen Ding, Leming Shi, Yuanting Zheng

**Affiliations:** 1 State Key Laboratory of Genetics and Development of Complex Phenotypes, Human Phenome Institute, School of Life Sciences, Shanghai Cancer Center, Fudan University, Shanghai, 200438 China; 2 International Human Phenome Institutes (Shanghai), Shanghai, 201210 China

The treatment efficacy of anti-diabetic therapies is highly heterogeneous among patients with type 2 diabetes (T2D) (Ahmad et al. 2022). Predictive biomarkers can be used to stratify patients into subgroups with varying efficacy before receiving the treatment, and help advance the understanding of disease and treatment (Ahmad et al. 2022). Thus, identifying predictive biomarkers is important for precision medicine of patients with T2D.

Approved in China in October 2021 as an adjunct to diet and exercise for improving glycemic control in adult patients with T2D, chiglitazar is a non-thiazolidinedione agonist of the α, δ and γ subtypes of the peroxisome proliferator-activated receptors (PPARs) (Deeks 2022). However, patients receiving chiglitazar also showed variable efficacy; that is, about 50% patients cannot achieve control of glycosylated hemoglobin type A1c (HbA1c) < 7% after 24-week treatment (Ji et al. 2021; Jia et al. 2021). To propose potential biomarkers for predicting chiglitazar efficacy, we studied the Chiglitazar perturbed Human multi-Omics ProfilE (ChiHOPE) cohort (http://fudan-pgx.org/chihope/, the details are described in our accompanying paper, which is currently under review), which was constructed from two phase three clinical trials of chiglitazar with 835 patients. To simulate a real-world clinical application scenario, the cohort was split into the derivation cohort (the first 50% of enrolled patients) and the temporal validation cohort (the later 50% of enrolled patients) (Jachs et al. 2024), enabling us to study how a marker or a model can be applied to predict treatment efficacy in future patients. The clinical characteristics between the derivation and validation cohorts showed no significant differences, suggesting that the two cohorts are distributionally comparable (Table S1).

In patients receiving chiglitazar treatment from the derivation cohort (*n* = 230), we identified robust predictive proteins associated with chiglitazar efficacy by stability selection based on Lasso logistic regression (Meinshausen and Bühlmann 2010). Glutathione peroxidase 3 (GPX3) emerged as the most frequently selected predictive protein for chiglitazar efficacy during 100 subsamples generated via the multi-split bootstrap, indicating its potential to predict chiglitazar efficacy (Fig. 1a, Table S2). Specifically, patients with lower baseline GPX3 levels showed better efficacy after 24 weeks of chiglitazar treatment (Fig. 1b, Fig. S1a). We evaluated the internal predictive performance of GPX3 using 10-times 5-fold cross-validation. By averaging the predicted probabilities across all folds for each sample, we obtained a cross-validated area under the ROC curve (AUC) of 0.655, indicating a moderate and robust predictive ability (Fig. 1c, Table S3). In the validation cohort (*n* = 217), although the performance of GPX3 was attenuated (AUC = 0.577, Table S4), the consistent direction supported GPX3 as a potential predictive biomarker (Fig. 1b, Fig. S1b).

Previous research has proposed several clinical markers predictive for chiglitazar efficacy, including BMI, fasting insulin, HbA1c, HDL-C and sex (Huang et al. 2023). We compared the predictive performance of GPX3 to previously proposed markers and other important clinical variables. Among all variables investigated, GPX3 showed better or comparable predictive performance than previous proposed markers, while lower predictive performance than glucose-related traits including 2-hour glucose, fasting glucose and HOMA-B (Fig. 1c, Fig. S2, Tables S3-S6).

However, different from the glucose-related traits (Fig. 1d, Fig. S3), the predictive ability of GPX3 for therapeutic efficacy is specific to chiglitazar, suggesting its potential utility in guiding precision therapy (Fig. 1d). To evaluate whether the predictive ability of GPX3 to treatment efficacy varied between chiglitazar and sitagliptin, we studied the patients treated by chiglitazar and sitagliptin from the clinical trials, and compared a logistic regression model including only the main effects of GPX3 and treatment group with a model that also included their interaction. Adding the interaction term significantly improved model fit (likelihood ratio test, *p* = 0.008). Specifically, compared to the sitagliptin group, lower plasma GPX3 level is associated with better efficacy in the chiglitazar group, suggesting the potential ability for GPX3 to guide choice between chiglitazar and sitagliptin (Table S7). In addition, patients with chiglitazar efficacy also showed a significant increase in GPX3 after 24-week of chiglitazar treatment (*p* = 0.006, Fig. 1d), and the magnitude of GPX3 elevation was associated with a greater reduction in fasting glucose (*p* = 0.032, Fig. S4), suggesting both the baseline level and treatment-induced change in GPX3 may be associated with glycemic improvement during chiglitazar therapy.

Previous in vivo and in vitro experiments have demonstrated that GPX3 plays a protective role against insulin resistance, inflammation and adipose dysfunction (Hauffe et al. 2020; Qi et al. 2018; Song et al. 2024). In preadipocytes, *GPX3* knock out impairs insulin sensitivity, whereas *GPX3* overexpression enhances insulin receptor expression and improves adipocyte differentiation and function (Hauffe et al. 2020). Consistently, in drug-naïve T2D patients from the ChiHOPE cohort, lower baseline plasma GPX3 levels were correlated with more adverse metabolic profiles, including lower high-density lipoprotein cholesterol (HDL-C), and higher insulin resistance, circulating lipids and obesity (Fig. 2a, Table S8). These correlations were replicated in the independent National Survey of Physical Traits (NSPT) cohort (Zhang et al. 2022) (Fig. 2b, Table S9). In addition, in the adipose tissue where *GPX3* is highly expressed (Zhong et al. 2025), lower *GPX3* expression was correlated with insulin resistance, obesity, inflammation and circulating levels of lipids and glucose (Fig. 2c). Besides, previous studies have shown that *GPX3* is required for the regulation of PPARγ-mediated antioxidant effects (Chung et al. 2009; Reddy et al. 2018). Given that PPARγ is one of the molecular targets of chiglitazar, these findings suggest that GPX3 may contribute to the therapeutic effects of chiglitazar through its involvement in PPARγ signaling.

The potential clinical utility of GPX3 as a biomarker lies in its ability to help precision medicine in T2D, that patients with lower GPX3 could benefit more from the treatment of chiglitazar than sitagliptin. If validated prospectively, GPX3 could be measured from baseline serum samples using widely available immunoassay techniques, enabling clinicians to identify patients more likely to benefit from chiglitazar. This would facilitate a more tailored approach to therapy and potentially improve treatment efficiency by avoiding ineffective medications. In the future, GPX3 might be incorporated into clinical decision algorithms alongside other biomarkers or clinical parameters to inform personalized diabetes management. However, before implementation, standardization of GPX3 measurement, cost-effectiveness assessment, and integration into clinical workflows will be essential.

Our study has several limitations. First, this study was based on a retrospective analysis of clinical trials, and prospective validation in larger and more diverse populations is necessary to confirm its predictive value in guiding clinical treatment decisions, such as choosing between chiglitazar and sitagliptin. Second, similar to other biomarkers proposed for predicting treatment response in T2D, the predictive power of GPX3 alone was moderate. Future studies are needed to explore whether integrating GPX3 with other molecular or clinical features could improve the accuracy and clinical utility of response prediction. Third, we only showed the correlations between GPX3 and T2D phenotypes. Further experimental work is required to investigate the underlying biological mechanisms and to determine whether GPX3 is also involved in the response to other PPAR agonists.

Together, our findings highlight GPX3 as a promising biomarker for treatment stratification in T2D, and lay the groundwork for its future clinical validation and implementation in personalized therapy.

**Fig. 1:**
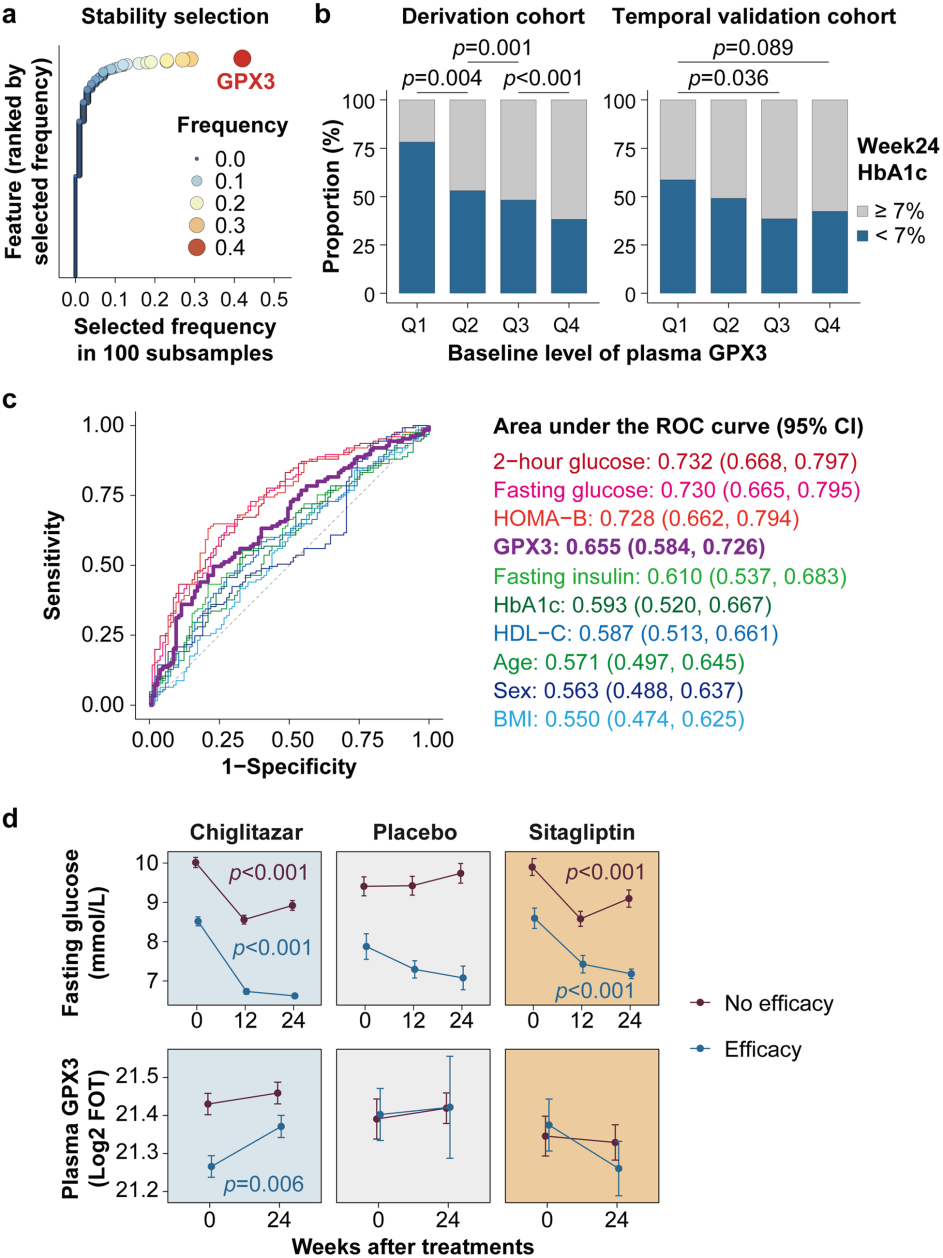
Baseline Plasma GPX3 Level Predicts Efficacy of Insulin-Sensitization Drug Chiglitazar in Type 2 Diabetes. **a**, The selected frequency of features during 100 subsamples in the derivation cohort. **b**, Proportions of efficacy in patients with different baseline plasma GPX3 levels receiving chiglitazar treatment, from the lowest (Q1) to the highest (Q4). **c**, ROC curves for baseline variables to predict chiglitazar efficacy in the derivation cohort. **d**, Fasting glucose and plasma GPX3 levels in patients with and without efficacy in different treatment groups (mean ± SE)

**Fig. 2:**
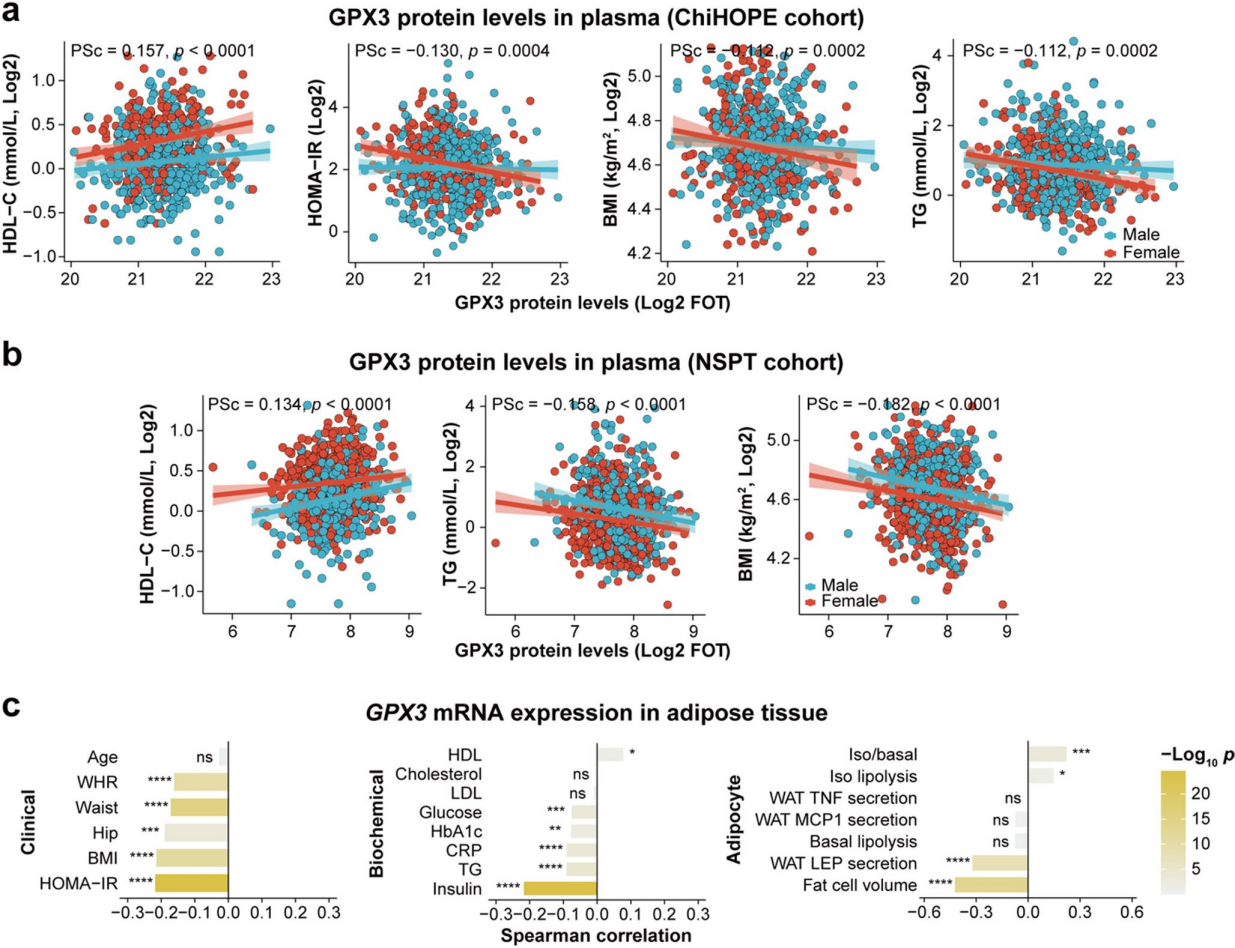
Correlations between GPX3 and T2D phenotypes. **a**, Correlations between plasma GPX3 levels and T2D phenotypes in > 700 drug-naïve patients from the ChiHOPE cohort. **b**, Correlations between plasma GPX3 levels and T2D phenotypes in > 1000 individuals from the NSPT cohort. **c**, Correlations between *GPX3* expression in adipose tissue and T2D phenotypes from the adiposetissue knowledge portal (https://adiposetissue.org/). *p < 0.05, **p < 0.01, ***p < 0.001, ***p < 0.0001. BMI: body mass index; ChiHOPE: Chiglitazar perturbed Human multi-Omics ProfilE; CRP: C-reactive protein; FOT: fraction of total; GPX3: glutathione peroxidase 3; HDL-C: high density lipoprotein cholesterol; HOMA-IR: homeostatic model assessment of insulin resistance; LEP: leptin; MCP1: monocyte chemoattractant protein-1; NSPT: National Survey of Physical Traits; PSc: partial Spearman’s rank correlation coefficient; TG: triglyceride; TNF: tumor necrosis factor; WAT: white adipose tissue; WHR: waist-to-hip ratio

## Electronic Supplementary Material

Below is the link to the electronic supplementary material.

Supplementary Material 1

## Data Availability

The proteomic and genomic datasets of the ChiHOPE cohort have been deposited at National Omics Data Encyclopedia (NODE, https://www.biosino.org/node) and are publicly available through the accession numbers OEP00002924. The correlation data in Fig. 2c can be accessed at the adiposetissue knowledge portal (https://adiposetissue.org/).
