# Supplementary material for: Baseline Plasma GPX3 Level Predicts Efficacy of Insulin-Sensitization Drug Chiglitazar in Type 2 Diabetes
Source: Phenomics. 2025 Jul 29;5(3):338–42. doi: 10.1007/s43657-025-00266-1 (PMC12391574; doi:10.1007/s43657-025-00266-1)
Supplement: Supplementary file 1 — Supplementary Material 1 [file 43657_2025_266_MOESM1_ESM.docx]

**Supplementary Materials**

**Supplementary Figures**


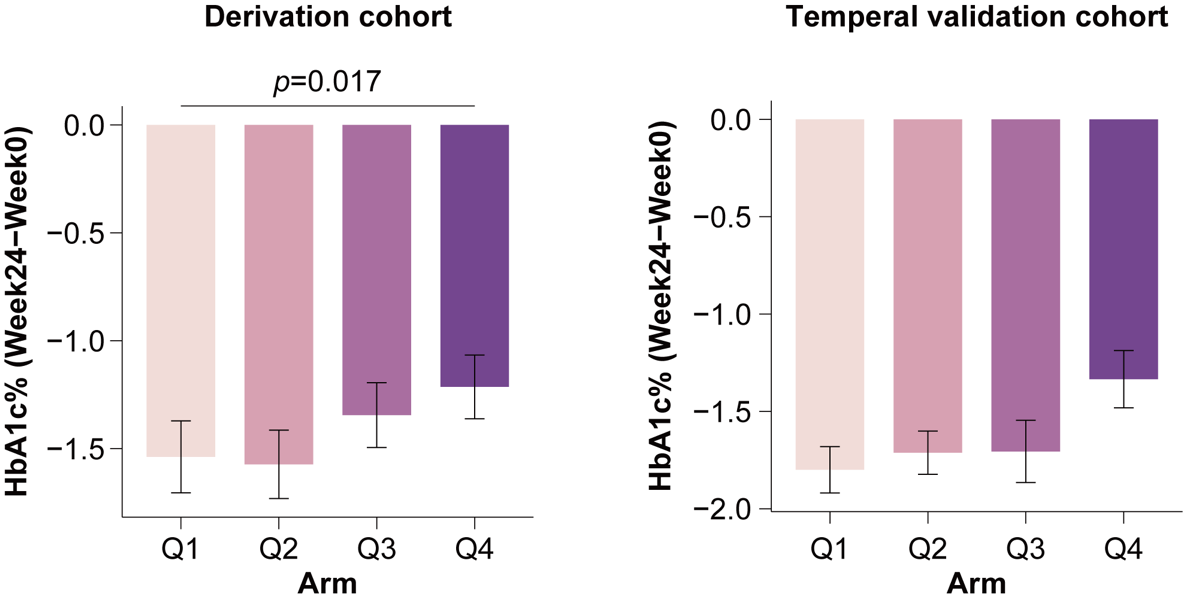


**Fig. S1 Baseline level of plasma GPX3 predicts chiglitazar efficacy**

Patients were stratified based on their baseline plasma GPX3 levels, from the lowest (Q1) to the highest (Q4). The y axis shows the change of HbA1c (%) after 24 weeks of chiglitazar treatment (mean ± SE)


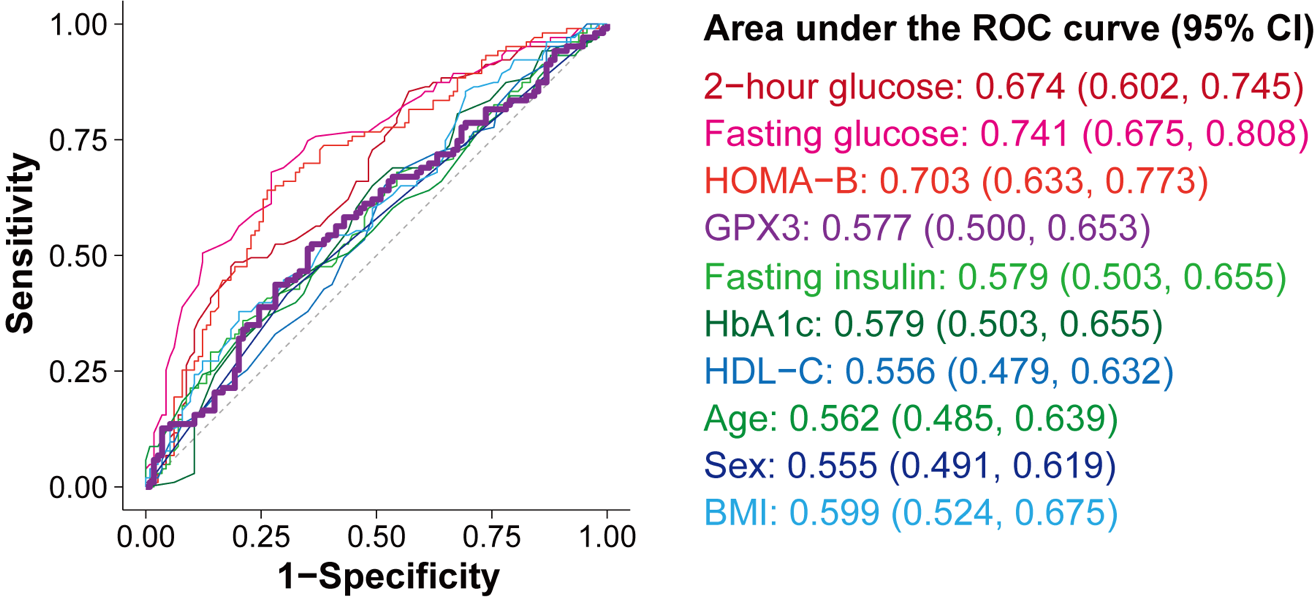


**Fig. S2 ROC curves for baseline variables to predict chiglitazar efficacy in the temporal validation cohort**


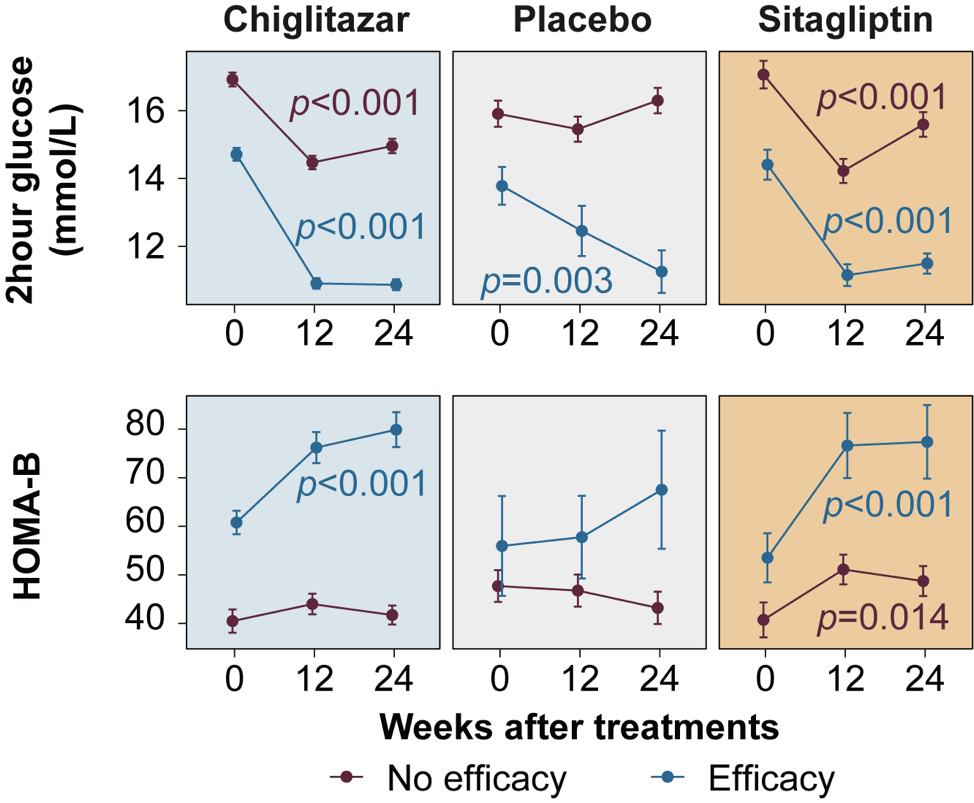


**Fig. S3 Two-hour glucose and HOMA-B levels in patients with and without efficacy in different treatment groups (mean ± SE)**

**
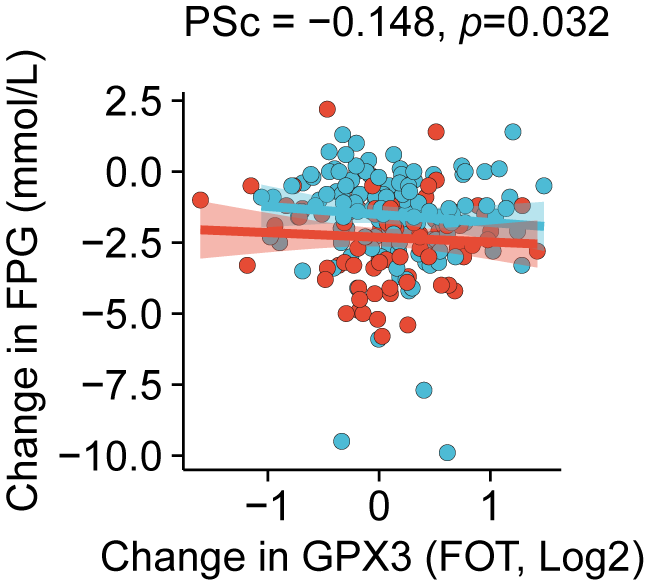
**

**Fig. S4 Correlations between changes in plasma GPX3 and fasting glucose after being treated with chiglitazar for 24 weeks, adjusting for sex, age, eGFR and trial**

PSc, partial Spearman’s correlation coefficient

**Supplementary Tables**

**Table S1 Baseline clinical characteristics of patients with type 2 diabetes in the derivation and temporal validation cohorts**

| **Clinical analytes** | **Derivation cohort** | | | **Temporal validation cohort** | | | ***p* value** |
| --- | --- | --- | --- | --- | --- | --- | --- |
|  | **n** | **mean** | **s.d.** | **n** | **mean** | **s.d.** |  |
| ***Basic*** |  |  |  |  |  |  |  |
| Age (yrs) | 240 | 78.88 | 9.55 | 239 | 78.28 | 9.31 | 0.269 |
| Diastolic blood pressure (mmHg) | 240 | 125.53 | 14.51 | 239 | 125.54 | 14.17 | 0.958 |
| Systolic blood pressure (mmHg) | 240 | 26.06 | 3.2 | 239 | 26.02 | 3.26 | 0.747 |
| BMI (kg/m^2^) | 240 | 72.48 | 11.92 | 239 | 71.9 | 11.37 | 0.748 |
| Weight (kg) | 240 | 91.62 | 9.21 | 239 | 91.36 | 8.57 | 0.793 |
| Waist circumstance (cm) | 240 | 78.88 | 9.55 | 239 | 78.28 | 9.31 | 0.269 |
| ***Glucose & insulin*** |  |  |  |  |  |  |  |
| HbA1c (%) | 240 | 8.64 | 0.72 | 239 | 8.57 | 0.7 | 0.318 |
| Fasting glucose (mmol/L) | 240 | 9.34 | 2.12 | 239 | 9.14 | 1.9 | 0.578 |
| 2-hour glucose (mmol/L) | 240 | 15.83 | 3.44 | 239 | 15.72 | 3.01 | 0.952 |
| Fasting insulin (mU/L) | 240 | 12.65 | 8.42 | 239 | 13.57 | 9.07 | 0.151 |
| HOMA-B | 238 | 48.01 | 34.61 | 239 | 53.98 | 42.13 | 0.131 |
| HOMA-IR | 238 | 5.23 | 4.08 | 239 | 5.53 | 4.02 | 0.269 |
| ***Lipid (mmol/L)*** |  |  |  |  |  |  |  |
| Triglyceride | 239 | 2.05 | 1.94 | 239 | 2.15 | 1.42 | 0.056 |
| Free fatty acids | 240 | 0.51 | 0.19 | 239 | 0.49 | 0.19 | 0.110 |
| Total cholesterol | 239 | 4.86 | 0.91 | 239 | 4.79 | 0.92 | 0.585 |
| HDL cholesterol | 239 | 1.15 | 0.27 | 239 | 1.13 | 0.28 | 0.533 |
| LDL cholesterol | 239 | 2.81 | 0.76 | 239 | 2.71 | 0.83 | 0.154 |
| ***Protein (Log2 FOT)*** |  |  |  |  |  |  |  |
| GPX3 | 233 | 21.35 | 0.44 | 217 | 21.34 | 0.43 | 0.890 |

The *p* values are calculated by the Wilcoxon Rank Sum test between the derivation and temporal validation cohorts

**Table S2 Features with selected frequency larger than 0.10 during 100 times of subsampling in the training dataset**

| **Feature** | **Selected frequency** |
| --- | --- |
| GPX3 | 0.42 |
| APOA2 | 0.29 |
| IGKV1-17 | 0.27 |
| KRT2 | 0.23 |
| SELENOP | 0.23 |
| MSN | 0.19 |
| FGG | 0.18 |
| COL6A3 | 0.16 |
| MASP2 | 0.13 |
| ACACA | 0.12 |
| APOL1 | 0.12 |
| STXBP5 | 0.12 |
| PON1 | 0.11 |

**Table S3 Performance of single indicators in the derivation set**

| **Variables** | **TP** | **TN** | **FP** | **FN** | **AUC** | **Precision** | **Sensitivity** | **Specificity** | **Accuracy** | **F1** | **MCC** |
| --- | --- | --- | --- | --- | --- | --- | --- | --- | --- | --- | --- |
| GPX3 | 96 | 48 | 57 | 29 | 0.655 | 0.627 | 0.768 | 0.457 | 0.626 | 0.691 | 0.238 |
| HbA1c | 88 | 46 | 59 | 37 | 0.593 | 0.599 | 0.704 | 0.438 | 0.583 | 0.647 | 0.147 |
| FPG | 101 | 54 | 51 | 24 | 0.730 | 0.664 | 0.808 | 0.514 | 0.674 | 0.729 | 0.339 |
| PBG | 92 | 62 | 43 | 33 | 0.732 | 0.681 | 0.736 | 0.590 | 0.670 | 0.708 | 0.330 |
| FI | 84 | 50 | 55 | 41 | 0.610 | 0.604 | 0.672 | 0.476 | 0.583 | 0.636 | 0.151 |
| HOMA-B | 81 | 72 | 33 | 44 | 0.728 | 0.711 | 0.648 | 0.686 | 0.665 | 0.678 | 0.332 |
| eGFR | 102 | 30 | 75 | 23 | 0.559 | 0.576 | 0.816 | 0.286 | 0.574 | 0.675 | 0.120 |
| HDL-C | 94 | 33 | 72 | 31 | 0.587 | 0.566 | 0.752 | 0.314 | 0.552 | 0.646 | 0.074 |
| BMI | 99 | 28 | 77 | 26 | 0.550 | 0.562 | 0.792 | 0.267 | 0.552 | 0.658 | 0.069 |
| Sex | 125 | 0 | 105 | 0 | 0.563 | 0.543 | 1.000 | 0.000 | 0.543 | 0.704 | NA |
| Age | 93 | 39 | 66 | 32 | 0.571 | 0.585 | 0.744 | 0.371 | 0.574 | 0.655 | 0.124 |

TP: true positive; TN: true negative; FP: false positive; FN: false negative; AUC: area under the receiver operating characteristic curve; MCC: Matthew’s correlation coefficient; FI: fasting insulin

**Table S4 Performance of single indicators in the temporal validation set**

| **Variables** | **TP** | **TN** | **FP** | **FN** | **AUC** | **Precision** | **Sensitivity** | **Specificity** | **Accuracy** | **F1** | **MCC** |
| --- | --- | --- | --- | --- | --- | --- | --- | --- | --- | --- | --- |
| GPX3 | 71 | 45 | 69 | 32 | 0.577 | 0.507 | 0.689 | 0.395 | 0.535 | 0.584 | 0.088 |
| HbA1c | 76 | 39 | 75 | 27 | 0.579 | 0.503 | 0.738 | 0.342 | 0.530 | 0.598 | 0.087 |
| FPG | 79 | 59 | 55 | 24 | 0.741 | 0.590 | 0.767 | 0.518 | 0.636 | 0.667 | 0.292 |
| PBG | 77 | 55 | 59 | 26 | 0.674 | 0.566 | 0.748 | 0.482 | 0.608 | 0.644 | 0.237 |
| FI | 75 | 38 | 76 | 28 | 0.579 | 0.497 | 0.728 | 0.333 | 0.521 | 0.591 | 0.067 |
| HOMA-B | 74 | 71 | 43 | 29 | 0.703 | 0.632 | 0.718 | 0.623 | 0.668 | 0.673 | 0.342 |
| eGFR | 79 | 28 | 86 | 24 | 0.562 | 0.479 | 0.767 | 0.246 | 0.493 | 0.590 | 0.015 |
| HDL-C | 79 | 33 | 81 | 24 | 0.556 | 0.494 | 0.767 | 0.289 | 0.516 | 0.601 | 0.064 |
| BMI | 90 | 27 | 87 | 13 | 0.600 | 0.508 | 0.874 | 0.237 | 0.539 | 0.643 | 0.142 |
| Sex | 103 | 0 | 114 | 0 | 0.555 | 0.475 | 1.000 | 0.000 | 0.475 | 0.644 | NA |
| Age | 80 | 31 | 83 | 23 | 0.562 | 0.491 | 0.777 | 0.272 | 0.512 | 0.602 | 0.056 |

TP: true positive; TN: true negative; FP: false positive; FN: false negative; AUC: area under the receiver operating characteristic curve; MCC: Matthew’s correlation coefficient; FI: fasting insulin

**Table S5 DeLong’s test between the AUC of GPX3 with other variables in the derivation set**

| **Variable** | ***p* value** |
| --- | --- |
| HbA1c | 2.36E-01 |
| FPG | 1.50E-01 |
| PBG | 1.22E-01 |
| FI | 3.46E-01 |
| HOMA-B | 1.33E-01 |
| eGFR | 6.59E-02 |
| HDL-C | 1.86E-01 |
| BMI | 3.30E-02 |
| Sex | 1.43E-05 |
| Age | 1.11E-01 |

**Table S6 DeLong’s test between the AUC of GPX3 with other variables in the temporal validation set**

| **Variable** | ***p* value** |
| --- | --- |
| HbA1c | 9.62E-01 |
| FPG | 1.27E-03 |
| PBG | 6.83E-02 |
| FI | 9.63E-01 |
| HOMA-B | 1.11E-02 |
| eGFR | 7.84E-01 |
| HDL-C | 6.96E-01 |
| BMI | 6.51E-01 |
| Sex | 6.52E-01 |
| Age | 7.89E-01 |

**Table S7 Summary statistics of variables and their interactions with treatment groups to predict efficacy**

| **GPX3, likelihood ratio test, *p* = 0.008** | | | | |
| --- | --- | --- | --- | --- |
|  | **Estimate** | **Std. Error** | **z value** | ***p* (>\|z\|)** |
| armCG | 22.825 | 8.582 | 2.660 | 0.008 |
| GPX3 | 0.112 | 0.327 | 0.341 | 0.733 |
| armCG:GPX3 | -1.058 | 0.402 | -2.633 | 0.008 |
| **HBA1C, likelihood ratio test, *p* = 0.492** | | | | |
|  | **Estimate** | **Std. Error** | **z value** | ***p* (>\|z\|)** |
| armCG | 1.863 | 2.353 | 0.792 | 0.429 |
| HBA1C | -0.266 | 0.236 | -1.128 | 0.259 |
| armCG:HBA1C | -0.188 | 0.273 | -0.690 | 0.490 |
| **FPG, likelihood ratio test, *p* = 0.161** | | | | |
|  | **Estimate** | **Std. Error** | **z value** | ***p* (>\|z\|)** |
| armCG | 1.682 | 1.022 | 1.646 | 0.100 |
| FPG | -0.294 | 0.091 | -3.220 | 0.001 |
| armCG:FPG | -0.157 | 0.110 | -1.427 | 0.154 |
| **PBG, likelihood ratio test, *p* = 0.427** | | | | |
|  | **Estimate** | **Std. Error** | **z value** | ***p* (>\|z\|)** |
| armCG | 1.054 | 1.019 | 1.035 | 0.301 |
| PBG | -0.202 | 0.052 | -3.879 | 0.000 |
| armCG:PBG | -0.051 | 0.064 | -0.802 | 0.422 |
| **FI, likelihood ratio test, *p* = 0.138** | | | | |
|  | **Estimate** | **Std. Error** | **z value** | ***p* (>\|z\|)** |
| armCG | -0.191 | 0.338 | -0.566 | 0.572 |
| FINS | 0.000 | 0.019 | 0.026 | 0.979 |
| armCG:FI | 0.034 | 0.023 | 1.488 | 0.137 |
| **HOMA-B, likelihood ratio test, *p* = 0.261** | | | | |
|  | **Estimate** | **Std. Error** | **z value** | ***p* (>\|z\|)** |
| armCG | -0.132 | 0.345 | -0.383 | 0.702 |
| HOMA.B | 0.010 | 0.005 | 1.919 | 0.055 |
| armCG:HOMA-B | 0.007 | 0.006 | 1.162 | 0.245 |
| **eGFR, likelihood ratio test, *p* = 0.335** | | | | |
|  | **Estimate** | **Std. Error** | **z value** | ***p* (>\|z\|)** |
| armCG | 1.267 | 1.048 | 1.209 | 0.227 |
| EGFR | -0.002 | 0.010 | -0.173 | 0.863 |
| armCG:EGFR | -0.010 | 0.011 | -0.967 | 0.333 |
| **HDL-C, likelihood ratio test, *p* = 0.343** | | | | |
|  | **Estimate** | **Std. Error** | **z value** | ***p* (>\|z\|)** |
| armCG | 0.925 | 0.752 | 1.230 | 0.219 |
| HDLC | -0.422 | 0.519 | -0.813 | 0.416 |
| armCG:HDLC | -0.600 | 0.630 | -0.952 | 0.341 |
| **BMI, likelihood ratio test, *p* = 0.030** | | | | |
|  | **Estimate** | **Std. Error** | **z value** | ***p* (>\|z\|)** |
| armCG | -3.232 | 1.606 | -2.012 | 0.044 |
| BMI | -0.044 | 0.054 | -0.801 | 0.423 |
| armCG:BMI | 0.134 | 0.062 | 2.162 | 0.031 |
| **Age, likelihood ratio test, *p* = 0.509** | | | | |
|  | **Estimate** | **Std. Error** | **z value** | ***p* (>\|z\|)** |
| armCG | -0.425 | 1.040 | -0.409 | 0.683 |
| Age | 0.014 | 0.017 | 0.809 | 0.418 |
| armCG:Age | 0.013 | 0.020 | 0.662 | 0.508 |
| **Sex, likelihood ratio test, *p* = 0.245** | | | | |
|  | **Estimate** | **Std. Error** | **z value** | ***p* (>\|z\|)** |
| armCG | 0.081 | 0.236 | 0.346 | 0.730 |
| SexFemale | -0.065 | 0.355 | -0.184 | 0.854 |
| armCG:SexFemale | 0.472 | 0.407 | 1.159 | 0.246 |

**Table S8 Partial Spearman’s correlation between plasma GPX3 levels and T2D phenotypes in drug-naïve patients with T2D from the ChiHOPE cohort, adjusting for age, sex and eGFR**

| **Phenotypes** | **#Individuals** | **Estimate** | **Statistic** | ***p* value** |
| --- | --- | --- | --- | --- |
| HDL-C | 734 | 0.157 | 4.287 | 2.05E-05 |
| Fasting insulin | 735 | -0.140 | -3.819 | 1.45E-04 |
| HOMA-IR | 729 | -0.130 | -3.525 | 4.50E-04 |
| BMI | 735 | -0.112 | -3.054 | 2.34E-03 |
| TG | 734 | -0.112 | -3.032 | 2.51E-03 |
| ALT | 735 | -0.110 | -2.990 | 2.89E-03 |
| HOMA-B | 729 | -0.109 | -2.937 | 3.41E-03 |
| hsCRP | 668 | -0.106 | -2.753 | 6.07E-03 |
| Weight | 735 | -0.098 | -2.647 | 8.30E-03 |
| Serum creatinine | 735 | 0.081 | 2.206 | 2.77E-02 |
| AST | 735 | -0.081 | -2.196 | 2.84E-02 |
| Blood urea nitrogen | 735 | 0.080 | 2.180 | 2.95E-02 |
| 2-hour glucose | 733 | 0.080 | 2.160 | 3.11E-02 |
| Waist circumference | 735 | -0.077 | -2.091 | 3.69E-02 |

**Table S9** **Partial Spearman’s correlation between plasma GPX3 levels and T2D phenotypes in individuals from the NSPT cohort, adjusting for age and sex**

| **Phenotypes** | **#Individuals** | **Estimate** | **Statistic** | ***p* value** |
| --- | --- | --- | --- | --- |
| BMI | 1024 | -0.182 | -5.899 | 4.96E-09 |
| TG | 1011 | -0.158 | -5.081 | 4.46E-07 |
| HDL-C | 1011 | 0.134 | 4.301 | 1.87E-05 |
| LDL-C | 1011 | -0.066 | -2.092 | 3.66E-02 |

**Methods**

**Study population**

***The ChiHOPE cohort***

The ChiHOPE cohort of 835 participants was constructed from drug-naïve patients with T2D recruited from two multi-center, randomized, double-blind phase 3 trials of chiglitazar (CMAP, placebo-controlled, ClinTrials.gov registration no. NCT02121717; CMAS, sitagliptin-controlled, ClinTrials.gov registration no. NCT02173457). The inclusion criteria were described in detail elsewhere (Ji et al. 2021; Jia et al. 2021). Briefly, patients with previously untreated recent-onset T2D were eligible for inclusion, if they were aged 18-70 years with a BMI range of 18.5-35.0 kg/m^2^, and with insufficient glycemic control (HbA1c ≥ 58.5 mmol/mol [7.5%] and ≤ 85.8 mmol/mol [10.0%]) despite a strict diet and exercise regimen. Patients with T1D and other diseases were excluded.

***The NSPT cohort***

1,027 Han Chinese volunteers recruited from the Chinese city Zhengzhou, Henan province by National Survey of Physical Traits (NSPT). NSPT is a sub-project of the National Science & Technology Basic Research Project approved by the Ethics Committee of Human Genetic Resources of the School of Life Sciences, Fudan University, Shanghai (14117) (Zhang et al. 2022).

**Derivation and validation datasets**

The study subjects consisted of 534 T2D patients from the ChiHOPE cohort who received insulin sensitizer treatment. The training set was constructed from the first 270 (50.6%) T2D patients enrolled at each trial center, while the validation set was constructed from the later 264 (49.4%) patients. Specifically, 240 and 239 T2D patients with 24-week HbA1c data were used as the derivation and validation sets, respectively. Baseline clinical characteristics of T2D patients in the derivation and validation sets are shown in **Table S1**.

**Feature selection**

The predictive endpoint was defined as whether HbA1c levels dropped below 53 mmol/mol (7%) after 24 weeks of insulin sensitizer treatment. Lasso regression with cross-validation was used to determine the optimal regularization parameter, and selected features with non-zero coefficients. Then, stability selection was conducted with 100 subsamples generated via the multi-split bootstrap, and the selection frequency for each variable was calculated.

**Evaluation of predictive ability of single marker**

To assess the internal predictive performance of candidate biomarkers, we applied 10-times repeated 5-fold cross-validation within the derivation cohort. Specifically, the dataset was randomly split into five folds, with each fold used once as a validation set while the remaining four folds served as the training set, and the entire procedure was repeated ten times using different random seeds. A total of 50 prediction rounds were obtained per sample. For each round, a logistic regression model was trained and the predicted probability of treatment efficacy was recorded. To evaluate the internal robustness, we calculated the mean predicted probability across all 50 folds for each patient and used it to construct the receiver operating characteristic (ROC) curve. The area under the ROC curve (AUC) was computed as a measure of discriminative performance. All frozen predictors were applied on the temporal validation cohort, and their respective AUC-ROC, true positive (TP), true negative (TN), false positive (FP), false negative (FN), precision, sensitivity/recall, specificity, accuracy, F1 score, Matthews correlation coefficient (MCC) values were evaluated with the functions roc, auc and confusionMatrix from the R package caret (6.0.94). The 95% CI of each test AUC was obtained with the DeLong’s test by the function roc.test of R package pROC (1.18.5).

**Association analyses**

Association analyses between GPX3 and clinical phenotypes were conducted using the *glm* function from the R package stats (v4.4.1). Linear regression models were applied (*family = gaussian*). Covariates comprising sex, age, eGFR, baseline HbA1c and clinical trial were included. The p values were adjusted using the BH method, and associations with BH-adjusted p < 0.05 were considered significant.

To evaluate whether the predictive value of GPX3 differed by treatment groups, we constructed two logistic regression models: a base model including the main effects of GPX3 and treatment group, and an extended model additionally including the GPX3-by-treatment interaction term. We then performed a likelihood ratio test (LRT) to compare the goodness-of-fit between the two models. The LRT was conducted by comparing the difference in residual deviance between the models, and statistical significance was determined using a chi-squared distribution with degrees of freedom equal to the difference in the number of parameters. A significant *p*-value indicated that the interaction term significantly improved model fit, supporting a differential predictive effect of GPX3 across treatments.

**Correlation analyses**

The Spearman’s rank correlation was calculated with the function corr.test of the R package psych v.2.1.6. For partial correlation analyses, the partial Spearman’s rank correlation was calculated with the function pcor of the R package ppcor v.1.1.

All statistical analyses were conducted in R version 4.2.2 (R Foundation for Statistical Computing, Vienna, Austria). Significance was regarded as a two-sided P value < 0.05 unless otherwise specified.

**References**

Ji L, Song W, Fang H et al. (2021) Efficacy and safety of chiglitazar, a novel peroxisome proliferator-activated receptor pan-agonist, in patients with type 2 diabetes: a randomized, double-blind, placebo-controlled, phase 3 trial (CMAP). Sci Bull (Beijing) 66(15):1571-1580. <https://doi.org/10.1016/j.scib.2021.03.019>

Jia W, Ma J, Miao H et al. (2021) Chiglitazar monotherapy with sitagliptin as an active comparator in patients with type 2 diabetes: a randomized, double-blind, phase 3 trial (CMAS). Sci Bull (Beijing) 66(15):1581-1590. <https://doi.org/10.1016/j.scib.2021.02.027>

Zhang M, Wu S, Du S et al. (2022) Genetic variants underlying differences in facial morphology in East Asian and European populations. Nat Genet 54(4):403-411. <https://doi.org/10.1038/s41588-022-01038-7>
